# Modest phenotypic improvements in ASA-deficient mice with only one UDP-galactose:ceramide-galactosyltransferase gene

**DOI:** 10.1186/1476-511X-5-21

**Published:** 2006-08-07

**Authors:** S Franken, D Wittke, JE Mansson, R D'Hooge, PP De Deyn, R Lüllmann-Rauch, U Matzner, V Gieselmann

**Affiliations:** 1 Department of Physiological Chemistry, University of Bonn, Germany; 2 Anatomisches Institut, Universität Kiel, Germany; 3 Institute of Clinical Neuroscience, Goteborg University, Sweden; 4 Laboratory of Biological Psychology, University of Leuven, Belgium; 5 Department of Biomedical Sciences and Department of Neurology/Memory Clinic, University of Antwerp, Belgium; 6 Institut für Physiologische Chemie, Rheinische-Friedrich-Wilhelms Universität, Nussallee 11, 53115, Bonn, Germany

## Abstract

**Background:**

Arylsulfatase A (ASA)-deficient mice are a model for the lysosomal storage disorder metachromatic leukodystrophy. This lipidosis is characterised by the lysosomal accumulation of the sphingolipid sulfatide. Storage of this lipid is associated with progressive demyelination. We have mated ASA-deficient mice with mice heterozygous for a non-functional allele of UDP-galactose:ceramide-galactosyltransferase (CGT). This deficiency is known to lead to a decreased synthesis of galactosylceramide and sulfatide, which should reduce sulfatide storage and improve pathology in ASA-deficient mice.

**Results:**

ASA-/- CGT+/- mice, however, showed no detectable decrease in sulfatide storage. Neuronal degeneration of cells in the spiral ganglion of the inner ear, however, was decreased. Behavioural tests showed small but clear improvements of the phenotype in ASA-/- CGT+/- mice.

**Conclusion:**

Thus the reduction of galactosylceramide and sulfatide biosynthesis by genetic means overall causes modest improvements of pathology.

## Background

Metachromatic leukodystrophy (MLD) is a lysosomal storage disease of humans, which is characterised by progressive demyelination. The underlying defect is the deficiency of arylsulfatase A (ASA), which results in the lysosomal accumulation of the sphingolipid 3-O-sulfogalactosylceramide (sulfatide) predominantly in brain, kidney and bile ducts [1]. In the typical late infantile form of the disease patients start to develop gait disturbances, ataxia and paresis between the ages of 18 to 24 months. The neurologic symptoms usually progress rapidly and finally patients die in a decerebrated state. The disease is currently untreatable. Since MLD naturally only occurs in humans a mouse model was generated by targeted disruption of the ASA gene [2]. These animals store sulfatide in the same tissues as humans, including oligodendrocytes, Schwann cells and various neurons [2-4]. The accumulation of sulfatide in the central nervous system steadily increases with progressing age of the animals. However, the ASA-deficient mice have a mild phenotype, since they do not show widespread demyelination in the central nervous system typical for patients. Except for oligodendrocytes sulfatide is also stored in certain neurons, which in some cases leads to neuronal degeneration. This is particularly evident in the inner ear. In between 6 to 12 months of age neurons of the spiral ganglion of the inner ear degenerate completely [5,6]. In addition, ASA-deficient mice show a decrease of the axonal diameter in peripheral nerves [2,7]. The mice develop progressive neurological symptoms, which are, however, not lethal. In summary, the pathological and behavioural studies performed so far suggest that the animals resemble early stages of human disease [2,3,8-10].

Sulfatide is synthesised by sulfation of its precursor galactosylceramide which is formed from ceramide by catalysis of the enzyme UDP-galactose:ceramide-galactosyltransferase (CGT). Mice deficient for this enzyme do not produce galactosylceramide nor sulfatide [11,12]. Surprisingly, they are still able to myelinate since they compensatorily synthesise glucosylceramide [12]. They develop various neurologic symptoms and die at the age of 2 to 3 months [11-14]. Heterozygous littermates having only one copy of the CGT gene were investigated up to the age of three months. They do not show any pathological phenotype but display reduced levels of galactosylceramide as well as sulfatide [12,13]. In these studies galactosylceramide was found to be reduced to about 70–80% and sulfatide to about 80% of normal, respectively.

To investigate whether reduction of galactosylceramide synthesis is an option to treat MLD, CGT-heterozygous animals were mated with ASA-deficient mice to obtain ASA-/- CGT+/- animals. The influence of CGT heterozygosity on the phenotype of ASA-deficient mice was examined.

## Results

### Brain lipid analysis of ASA-deficient mice heterozygous for a non-functional CGT allele

**Figure 1:**
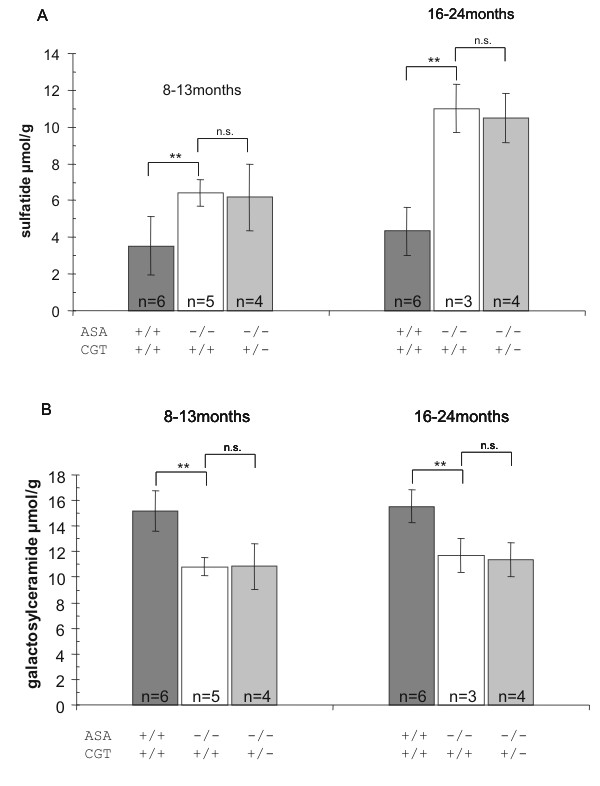
**Lipid analysis**. Sulfatide (A) and galactosylceramide (B) levels in whole brain extracts of ASA-deficient mice with one or two intact CGT genes and wild type controls. No significant difference between the ASA-deficient mice with different CGT backgrounds could be observed at all examined time points. Lipid concentrations are given in μmol per gram homogenized brain powder. Lipids were quantified by thin layer chromatography. The numbers of animals used for analysis (n) are given inside the columns. Error bars represent standard deviations of the mean values. **P ≤ 0.01 knock out against wild type by 1-way ANOVA; n.s.: not significant.

To investigate whether a decrease in galactosylceramide and sulfatide synthesis can improve the phenotype of ASA-deficient mice we crossed ASA-/- with CGT+/- mice. Genotypes of offspring at the ASA and CGT locus were determined by PCR as described under material and methods. Lipids were extracted from brains of mice with different genotypes and galactosylceramide and sulfatide were quantified by thin layer chromatography (see Figure 1). Compared to control animals (ASA+/+ CGT+/+) levels of sulfatide were increased in ASA-deficient animals but no significant difference was found between ASA-deficient mice with CGT+/+ and CGT+/- genetic background. Galactosylceramide levels are decreased in brains of MLD patients and ASA-deficient mice. This was also found in the mice investigated here and again no difference was found between CGT+/+ and CGT+/- mice. Thus, the alteration in CGT gene dosage has no obvious overall effect on sulfatide accumulation and galactosylceramide reduction in brains of ASA-deficient mice.

### Pathology of ASA-deficient mice heterozygous for a non-functional CGT allele

**Figure 2:**
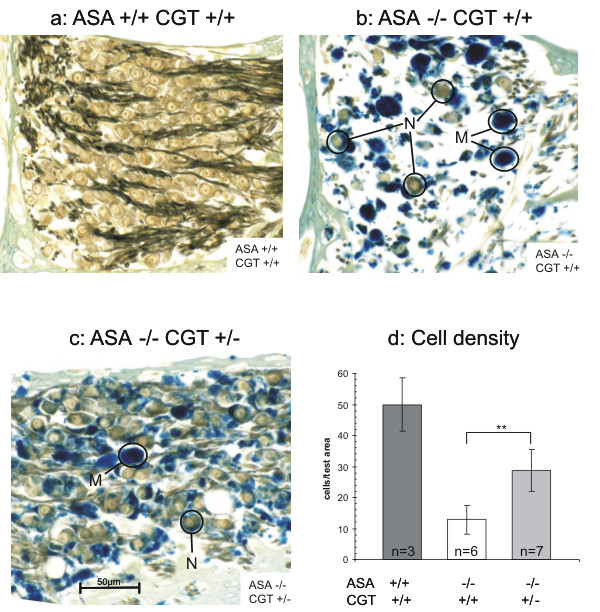
**Loss of neurons from the spiral ganglion of the inner ear**. Decalcified cochleae from mice of the indicated genotypes were incubated with alcian blue for selective staining of sulfatides, post-fixed with osmium tetroxide and embedded in paraffin. **a: ASA+/+ CGT+/+ mouse (age 18 months)**. The neuronal perikarya are densely packed and intermingled with myelinated fibres of the cochlear nerve. Alcianophilic (sulfatide rich) structures are absent from the ganglion. **b: ASA-/- CGT+/+ mouse (age 17 months)**. The neurons (N) contain alcianophilic material and are greatly reduced in number. The other cells which are heavily laden with alcianophilic material are macrophages (M) and Schwann cells (not labelled). **c: ASA-/- CGT+/- mouse (age 17 months)**. The loss of neurons is less dramatic than in c, whereas the sulfatide storage in neurons and macrophages appears similar. Bar, 50 μm for all figures. **d: Quantitative measurement**. Number of spiral ganglion perikarya per test area measuring 120 μm × 120 μm. Age of the deficient mice 14–17 months. Values for wild-type mice at 10–18 months of age were taken from [6]. The numbers of animals used for analysis (n) are given inside the columns. Error bars represent standard deviations of the mean values.**P ≤ 0.01 knock out against wild type by 1-way ANOVA.

Beside the nervous system kidneys of ASA-deficient mice show the highest amount of sulfatide storage material [15]. The histochemical examination of the kidneys revealed no differences between ASA-/- CGT +/- mice and ASA-/- CGT+/+ mice, as far as the degree and topographic distribution of sulfatide storage are concerned (results not shown). In the nervous system the most impressive degenerative alteration seen in ASA-deficient mice is the neuronal cell loss in the spiral ganglion of the inner ear starting 6 – 8 months after birth. At the age of 12 months the majority of neurons is lost in ASA-deficient mice [5,6]. To examine whether the heterozygosity for CGT has any influence on the pathology of ASA-deficient mice we quantified the cell loss in the spiral ganglion of mice with various genotypes. In these examinations ASA-deficient mice bearing only one intact CGT gene showed a clear improvement of pathology (see figure 2). The degeneration of neurons observed in the spiral ganglion of ASA-deficient mice was clearly mitigated in the CGT-heterozygous mice. This improvement can easily be seen by light microscopy (Fig. 2). We also quantified the number of cells by counting cells present in an area of defined size. This revealed a remarkable difference between ASA-/- CGT+/+ and ASA-/- CGT+/- mice. In animals older than 12 month only 26% of cells had survived in the spiral ganglion of ASA-deficient mice when compared to wild-type, whereas 57% were still structurally intact in mice bearing only one intact CGT gene (Figure 2d).

### Behavioural studies

**Figure 3:**
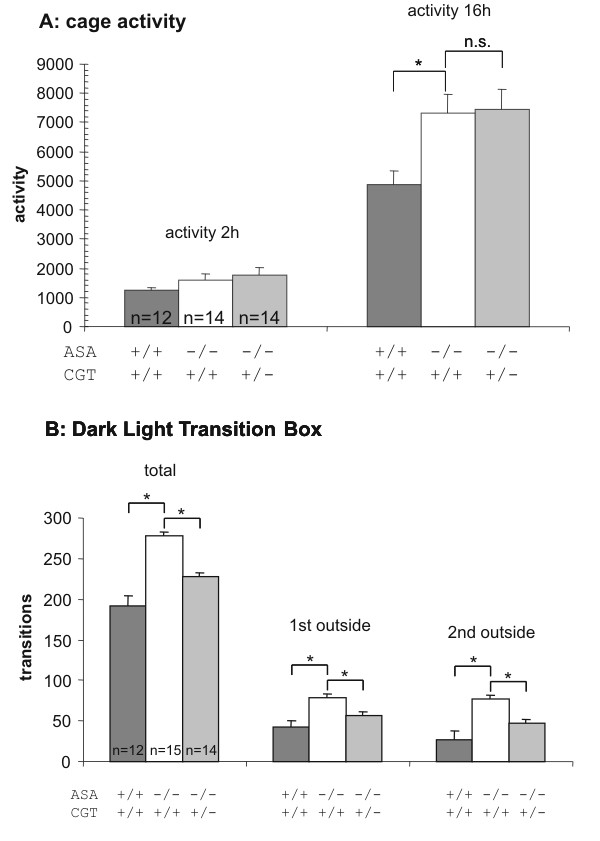
**Behavioural analysis of ASA-deficient mice with one or two intact CGT genes and wild type controls**. Wild type and ASA-deficient mice were tested like described. Whereas tests for cage activity (A) showed no improvement in ASA-deficient mice with only one intact CGT allele, the Dark-Light transition box test (B) showed a clear improvement in these animals. The numbers of animals used for analysis (n) are given inside the columns. Error bars represent standard deviations of the mean values. *P ≤ 0.05 knock out against wild type by 1-way ANOVA; n.s.: not significant.

ASA-deficient mice show various slowly progressing neurologic symptoms. The animals are hyperactive, have problems to stay on a rotating rod and show impairment in the morris water maze [10]. In the latter, however, impairments may be due to reduced neuromotor coordination capabilities rather than true learning deficits. To examine whether behavioural impairments are improved in ASA-/- CGT+/- mice, we performed various behavioural tests with these animals. When compared to wild-type and ASA-deficient mice no significant improvement in cage activity, rotarod or open field testing could be observed in ASA-deficient animals heterozygous for CGT. Only the Dark-Light-transition box test – a test of exploration and anxiety, but possibly less affected by the hyperactivity of the animals – showed a significant improvement in ASA-deficient mice bearing only one CGT allele (fig. 3).

## Discussion

In recent years various approaches to treat lysosomal storage diseases have been investigated. These include enzyme replacement therapy, gene therapy and substrate reduction therapy. Enzyme replacement therapy has been approved as therapy for various lysosomal storage diseases, such as Gaucher disease, Fabry disease, Pompe disease and certain kinds of mucopolysaccharidosis (for reviews see [16-20]). A number of reports deal with gene therapy approaches in animal models of lysosomal storage diseases [21-25]. However, although some of these studies show promising results the routine clinical application is not feasible in foreseeable future. In contrast, substrate reduction therapy has been shown to be a promising concept and is an approved therapy of some patient with Gaucher type 1 disease [26-31]. Compared to enzyme replacement, substrate reduction therapy may offer advantages for diseases with central nervous system involvement, because inhibition of synthesising enzymes depends on small molecule competitive inhibitors, which may be modified such that they can cross the blood brain barrier. In addition, treatment of hypercholesteremia with inhibitors of HMG-CoA-Reductase has proven that inhibition of lipid synthesis is a valuable clinical option to reduce levels of particular lipids [32,33]. The compound N-butyldeoxynojirimycin has been shown to inhibit the synthesis of glucosylceramide and is approved for therapy of Gaucher disease in which glucosylceramide accumulates [34]. Since glucosylceramide is also the precursor of gangliosides it is also employed in the treatment of GM_2 _gangliosidosis. The possible benefits of such a therapy has been demonstrated in animal models and clinical studies are already performed [31,35-40].

Whereas globoid cell leukodystrophy (Krabbe disease) is the result of defects in the degradation of galactosylceramide, sulfatide accumulation leads to MLD. Thus, the inhibition of either CGT or galactosylceramide sulfotransferase (CST) is therefore an option to reduce galactosylceramide and/or sulfatide levels in these lysosomal storage disorders. Galactosylceramide storing twitcher mice, a model for Krabbe disease, show a decreased life span and body weight, and they suffer from demyelination and various neurological symptoms [41-43]. To investigate the possible benefits of reduction of galactosylceramide synthesis for the treatment of Krabbe disease, twitcher mice were mated with mice bearing a deficient CGT allele to yield twitcher mice with only one copy of a functional CGT allele. Although the amount of galactosylceramide was not reduced in the CGT-heterozygous twitcher mice these animals showed a moderate but significant improvement of their phenotype [44].

In MLD sulfatide is the accumulating lipid, and patients suffer from demyelination and devastating neurologic symptoms [1]. Since the synthesis of sulfatide from ceramide is a two-step process both the enzymes involved – the CGT and the CST – are possible targets for inhibition of sulfatide synthesis. In contrast to glucosylceramide transferase, inhibitors of CGT or CST are currently not available. It was shown recently that mice bearing only one functional copy of the CGT gene have reduced levels of galactosylceramide as well as sulfatide [11,12]. Therefore we mated these animals with ASA-deficient mice. In the CGT-heterozygous ASA-deficient mice, however, the overall amount of sulfatide in brain was not reduced. In addition no effect on the reduction of galactosylceramide in ASA-deficient mice was seen in the animals heterozygous for CGT. This is in accordance with the results presented for CGT-heterozygous twichter mice [44]. Here also no reduction of galactosylceramide was found. In this study the authors interpreted this surprising results as coincidental. They assumed that the expected reduction in galactosylceramide is compensated by a prolonged survival of oligodendrocytes due to reduced galactosylceramide synthesis. As a consequence of reduced synthesis and preservation of oligodendrocytes the galactosylceramide levels appeared unaltered.

Beside lysosomal storage of sulfatide in myelin forming glia cells, examination of ASA-deficient mice has clearly shown, that also a number of neurons store sulfatide [2-4]. In neurons of the spiral ganglion this storage is accompanied with neuronal degeneration, whereas neuronal storage without any cellular damage can be observed in several nuclei of brainstem, diencephalon and spinal cord [2,4]. Thus, the pathogenesis of this lysosomal storage disorder beside its glial certainly also has a neuronal component. For the spiral ganglion we found that the manipulation of galactosylceramide and sulfatide biosynthesis leads to mitigation of neuronal degeneration, although ganglion cells still do store sulfatides (Fig. 2c). Former experiments have demonstrated that brain stem auditory-evoked potentials correlate with the amounts of neurons in the spiral ganglion [6]. Therefore we expect an improvement of the deafness phenotype seen in ASA-deficient mice. Unfortunately, due to the small number of neurons in this ganglion, we are not able to determine the extent of sulfatide storage at the cellular level, so that this improvement cannot directly be correlated with the accumulated amount of sulfatide.

## Conclusion

The behavioural studies revealed only very selective improvements in ASA-deficient CGT-heterozygous mice. Thus, the phenotypic and pathologic improvements in ASA-/- CGT+/- mice are significant but overall minor. If extrapolated to the clinical situation they are certainly not satisfactory. However, it should be taken into account that the reduction of sulfatide biosynthesis in CGT+/- mice is only around 20%. The results presented here suggest that a far more efficient inhibition is necessary to achieve a substantial positive clinical effect. Surprisingly, this slight reduction is obviously already sufficient to mitigate the neuronal degeneration in the ASA-deficient mice.

## Methods

### Chemicals

Silica gel 60 (230–400 mesh) and precoated HPTLC-plates were obtained from Merck AG, Darmstadt, Germany. The silica-gel plates used for TLC-ELISA were precoated plastic sheets, Polygram R. Sil G, from Macherey-Nagel, Düringen, Germany. Sulfatide and galactosylceramide standards were isolated and characterised at the Institute of Clinical Neuroscience, Goteborg University, Sweden.

### Generation of mice

Mice heterozygous for the CGT gene were a generous gift of B. Popko [12]. For generation of CGT +/+ and CGT +/- on wildtype and ASA-deficient background the CGT heterozygous (C57/Bl6) animals were bred with wildtype as well as ASA-deficient (both 129ola). CGT +/+ and +/- littermates on the different ASA backgrounds were used for analysis.

For the genotyping of animals DNA of mouse tail tips was analysed by PCR using the following primers: 5'-TGACCAAGGCCTTGTTCCCAT-3', 5'-TAGGGTGGAAGTTACCCTAGA-3', 5'-GGAGAGGCTATTCGGCTATGAC-3' for ASA genotyping and 5'-CTCTCAGAAGGCAGAGACATTGCC-3', 5'-CATCCATAGGCTGGACCCATGAAC-3', 5'-GGAGAGGCTATTCGGCTATGAC-3' for CGT genotyping;

The PCR program was as follows: initial denaturation at 94°C for 5 min, then 32 cycles 94°C 30 s, 56°C 60 s, 72°C 120 s.

For ASA PCR resulted in a 480 bp product for the wild-type allele and a 1.2 kb for the knockout allele and for CGT in a 558 bp and a 1.4 kb product, respectively.

### Lipid extraction and isolation

Frozen brains were mortared under liquid nitrogen and approximately 100 mg of the homogenised brain powder was processed for lipid extraction with chloroform:methanol:water, (C/M/W 4:8:3, by vol.) [45]. Phase partition was performed in C/M/0.1 M KCl (10:5:2, by vol.). The final lipid fraction, consisting of the lower phase, was stored in C/M/W (60:30:4,5 by vol.) and aliquots of each age were taken for sulfatide and galactosylceramide determination by densitometry after High Performance Thin Layer Chromatography and orcinol visualisation [46]. Significance of differences between means was assessed by 1-way ANOVA.

### Morphology

For the morphological examination animals 10 to 21 months of age were used. Some tissue samples of wild-type mice were taken from previous experiments. Under deep anaesthesia (intraperitoneal injection of tribromoethanol) the animals were killed by transcardial vascular perfusion with Bouin's mixture (diluted with phosphate-buffered saline to 25% of the original formula) or 6% glutaraldehyde in 0.1 M phosphate buffer (pH 7.4). Temporal bones were removed from the skull and decalcified with 4% EDTA [47]. Bones and thick slices of kidneys were processed for selective staining of sulfatides. This was achieved by incubation (3 weeks) with the cationic dye alcian blue (Alcec Blue, Aldrich, Steinheim, Germany) in the presence of 0.3 M MgCl_2 _as described [48]. The samples were then postfixed with 2% OsO_4 _for 3 hours and embedded in paraffin; sections (3–5 μm) were mounted with DePeX without further staining. Morphometric analysis of the spiral ganglion of the inner ear was performed by counting the ganglion cells in a test area measuring 120 μm × 120 μm. Significance of differences between means was assessed by 1-way ANOVA.

### Behavioural testing

Behavioural testings were performed as already described [8].

For cage activity recording mice are on a 12/12 h dark-light schedule (lights on at 8 AM, off at 8 PM). Standard transparent mouse cages (16 × 22 cm^2 ^bottom area; on sawdust bedding) are placed between 3 infrared photocells (2 along the short axis and 1 along the long axis of the cage), and the number of beam crossings is recorded during a defined period using a lab-built programmable counter. Single animals are placed in the recording cages in the late afternoon (dusk phase of their activity), and typically show an initial increase of exploratory activity followed by relatively high nocturnal activity. Activity is thus expressed as total beam crossings during the first 2 h as well as during the total 16 h overnight recording (4 PM through 8 AM).

Exploratory activity is assessed in the dark-light transition box. The box consists of a large illuminated compartment (55 × 73 cm^2^) and a smaller dark compartment (9 × 73 cm^2^) with four 4-cm holes, equally spaced in the dividing wall to allow transition between these compartments. Mice are first dark adapted for 1 h, and subsequently, placed in the dark compartment of the box. Exploratory activity is recorded for 10 min and expressed as the amount of crossings of 2 photobeams parallel to the dividing wall (beam 1: 1 cm from the wall; beam 2: 10 cm from the wall).

Significance of differences between means was assessed by 1-way ANOVA.

## Abbreviations

ASA, Arylsulfatase A; MLD, Metachromatic leukodystrophy; CGT, UDP-galactose:ceramide-galactosyltransferase; CST, galactosylceramide-sulfotransferase;

## Competing interests

The author(s) declare that they have no competing interests.

## Authors' contributions

SF was responsible for the mating of mice, designed the study, organized the collaboration and finalized the manuscript. DW and RLR carried out the histological experiment and participated in the design of the manuscript. JEM carried out the lipid analysis of brain samples. RD'H and PPDD performed the behavioral testings of the animals. UM and VG participated in the design and coordination of the study and helped to draft the manuscript. All authors read and approved the final manuscript.
